# Associations between inattention and impulsivity ADHD symptoms and disordered eating risk in a community sample of young adults

**DOI:** 10.1017/S0033291720004638

**Published:** 2022-10

**Authors:** E. Martin, C. T. Dourish, R. Hook, S. R. Chamberlain, S. Higgs

**Affiliations:** 1 School of Psychology, University of Birmingham, Birmingham, UK; 2 P1vital, Wallingford, Oxfordshire, UK; 3 Department of Psychiatry, University of Cambridge, Cambridge, UK; 4 Cambridgeshire & Peterborough NHS Foundation Trust, Cambridge, UK; 5 Department of Psychiatry, University of Southampton, Southampton, UK; 6 Southern Health NHS Foundation Trust, Southampton, UK

**Keywords:** Attention deficit hyperactivity disorder, disordered eating, hyperactivity, impulsivity, inattention, longitudinal

## Abstract

**Background:**

Symptoms of attention deficit hyperactivity disorder (ADHD) and trait impulsivity have been associated with disordered eating but are seldom assessed in community studies, or longitudinally and little is known about the mediating mechanisms.

**Methods:**

We tested associations between ADHD symptoms and disordered eating cross-sectionally and between trait impulsivity and disordered eating longitudinally. We utilised data from a normative cohort of young adults (642 participants: 65% female, *M*_age_ = 23 years). Participants were classified as high risk or low risk for disordered eating using the SCOFF instrument. In the first two steps of both cross-sectional and longitudinal hierarchical logistic regression models, demographics and covariates were entered. For the cross-sectional regression, Adult ADHD self-report scale (ASRS) scores, separated into inattentive and hyperactive/impulsive symptoms, were entered in the third step. In a separate longitudinal model, Barratt impulsivity scale subscales (attentional, motor and non-planning impulsivity) were entered in the third step. Depression, as assessed by the moods and feelings questionnaire (MFQ), was examined as a mediator.

**Results:**

Cross-sectionally, sex, MFQ score and inattentive symptoms predicted disordered eating risk (model *R*^2^ = 20%). Longitudinally, sex, MFQ score and attentional impulsivity predicted disordered eating risk (model *R*^2^ = 16%). The relationship between inattentive symptoms and the disordered eating risk was partially mediated by MFQ score, whereas the relationship between attentional impulsivity and the disordered eating risk was fully mediated by MFQ scores.

**Conclusions:**

These data highlight (1) a specific role for inattentive symptoms of ADHD and (2) the importance of both depression and impulsivity in predicting eating disorder risk.

## Introduction

Children and adults with symptoms of attention deficit hyperactivity disorder (ADHD) appear to be at higher risk of developing disordered eating (Kaisari, Dourish, & Higgs, 2017; Nazar et al., 2016). Evidence from longitudinal studies suggests that symptoms associated with ADHD may predict the onset of several types of eating disorders (Biederman et al., 2007; Rojo-Moreno et al., 2015; Viborg, Wångby-Lundh, & Lundh, 2014). For example, Sonneville et al. (2015) found that ADHD symptoms in childhood predicted binge eating behaviour during adolescence. Thus, these results suggest that features of ADHD may foster disordered eating behaviour.

To date, only a few studies have examined the relationship between specific ADHD symptoms and types of disordered eating. In addition, little is known about the underlying mechanisms and whether the relationship can be accounted for by variables known to be associated with both ADHD and disordered eating, e.g. negative mood. Seitz et al. (2013) reported that in females seeking treatment for bulimia nervosa (BN), the severity of BN was best predicted by inattentive symptoms, rather than impulsivity or hyperactivity symptoms of ADHD. On the other hand, data from a cross-sectional study of two independent adult samples suggested that both inattentive and hyperactive/impulsive ADHD symptoms are positively related to binge/disinhibited and restrictive eating, even when controlling for factors such as alcohol and drug use, other comorbid psychiatric disorders and medication (Kaisari, Dourish, Rotshtein, & Higgs, 2018). Negative mood was a mediator of several of these relationships, but in both studies, there was also a direct relationship between inattentive symptoms of ADHD and binge/disinhibited eating that was not fully accounted for by negative mood. Evidence from longitudinal studies is more limited, but Yilmaz et al. (2017) reported that a combination of inattentive and hyperactive/impulsive symptoms in children predicted higher eating disorder symptomatology during late adolescence, even when adjusting for levels of anxiety and depression. A study of younger children found that attention problems and hyperactivity were positively associated with prospective changes in food-related responses that may precede disordered eating (emotional overeating and satiety responsiveness) and that attention problems were also positively associated with changes in food responsiveness (Fuemmeler et al., 2020). Further research is required to clarify the relationship between inattentive and hyperactive/impulsive symptoms and disordered eating. In particular, because some types of disordered eating, e.g. binge eating disorder are more likely to emerge in later adolescence/early adulthood (Udo & Grilo, 2018) studies in young adults may shed more light on the potential significance of ADHD symptoms as risk factors. Peaks in the onset of eating disorders occur from teens to young adulthood, especially for BN and binge eating disorder where the average onset is later than that for anorexia nervosa (AN) but an eating disorder can emerge at any age (Christian et al., 2019). Young adulthood represents a major transition period as young adults launch into independence. Peer opinion becomes more important, and lifestyles are less structured and supervised and there is evidence for the emergence of eating disorders during this period (Pearson et al., 2017).

In addition, the broader concept of ‘trait’ self-report impulsivity is also highly relevant in this context. For example, a recent, large cross-sectional population-based study reported that trait impulsivity on the Barratt impulsivity scale (BIS) was associated with disordered eating (Bénard et al., 2019). In a systematic review, there was evidence from a range of studies that eating disorders were associated with significantly elevated impulsivity on self-report questionnaires (such as BIS) compared to controls (Waxman, 2009). This review highlighted several limitations in the corpus of literature: (i) the vast majority of research had been conducted only in women, (ii) studies using dimensional measures in more normative (population type) cohorts were lacking, and (iii) longitudinal studies were absent.

The current study had two primary aims. First, in a general population of young adults, we examined the cross-sectional relationship between inattentive and hyperactive/impulsive symptoms of ADHD and disordered eating behaviour (as assessed by the SCOFF eating disorder screening tool) while adjusting for factors which have been associated with both ADHD and disordered eating (e.g. alcohol or drug abuse and self-esteem). Based on previous work (e.g. Kaisari et al., 2017, 2018; Yilmaz et al., 2017), we predicted that both inattentive and hyperactive/impulsive symptoms would be positively associated with eating disorder risk. Second, we investigated the longitudinal relationships between trait impulsivity on the BIS, and subsequent disordered eating using data from baseline and follow up in the same data set. We hypothesised that the trait impulsivity would be positively associated with subsequent risk of eating disorder. Given our previous observations from cross-sectional studies (Kaisari et al., 2018) we also hypothesised that depression scores would mediate the predicted relationships between inattentive and hyperactive/impulsive ADHD symptoms and eating disorder risk.

## Methods

### Participants

This study used data from 642 young adults from the original cohort of the neuroscience in psychiatry network (NSPN) and who subsequently completed a follow-up survey in 2018–2019. NSPN is a previously established accelerated longitudinal study examining brain development in young people, which was designed to be representative of the general population; i.e. constitutes a normative cohort (Kiddle et al. (2018). The accelerated longitudinal design involves the recruitment of multiple, age-adjacent cohorts who are followed for a limited period. Each participant completed a home questionnaire pack (HQP) and sociodemographic questionnaire that focused on assessing participants' mood, behaviour and wellbeing along with demographic characteristics. Recruitment to the cohort was via general practitioners who were asked to recruit young people using their sex-age registers, schools and further education colleges and purposive advertisement. The HQP was sent to 3726 participants and returned by 65% of them (*N* = 2402, marking the baseline assessment stage of the NSPN 2400 Cohort). All the original participants who had previously provided baseline questionnaires via post (2012–2014) were contacted by email in 2018–2019 and asked to complete a follow up online survey implemented in SurveyMonkey (Chamberlain et al., 2019; Romero-Garcia et al., 2020). The time between baseline and follow-up assessments was mean of 3.85, s.d. of 0.41, and a range of 1–5. This survey examined a broader range of impulsivity/compulsivity measures than were included in the original study, some of which were not available at the time the NSPN cohort was conceived. Participants were excluded if they had a current or past history of clinical treatment for a psychiatric disorder, drug or alcohol dependence, neurological disorder including epilepsy, head injury causing loss of consciousness, or learning disability. Individuals taking psychotropic medication (including ADHD drugs) were also excluded from the study at the point of the cohort being established.

### Participant characteristics

Participants answered questions relating to socio-demographic characteristics (age, sex, and ethnicity) and other variables known to be associated with both ADHD and disordered eating: alcohol and drug use, depression and self-esteem.

### Measures of interest

The present study analysed measures from the baseline questionnaire and follow-up survey specifically relating to eating disorder risk and impulsivity and ADHD. Based on prior work we also selected specific variables known to be associated with both ADHD and eating disorders for inclusion as covariates: alcohol and drug use and depression and self-esteem measures.

### Alcohol use and drug use

Alcohol use was assessed using the fast alcohol screening test (FAST), which consists of four questions from the full alcohol use disorders identification test (Hodgson et al., 2003; Saunders, Aasland, Babor, De la Fuente, & Grant, 1993). Nicotine dependence was estimated using the Fagerström test for nicotine Dependence (FTND) (Heatherton, Kozlowski, Frecker, & Fagerstrom, 1991). The FTND has been used to test for nicotine dependence in both research and clinical settings and has good psychometric reliability (Pomerleau, Carton, Lutzke, Flessland, & Pomerleau, 1994). Illicit drug use was assessed using additional questions regarding the type of illicit drug(s) used and frequency in the last month.

### Self esteem

Self-esteem was assessed using the ten-item Rosenberg self-esteem scale (RSE; Rosenberg, 1965). Items are rated on a four-point Likert scale, ranging from (1) strongly disagree to (4) strongly agree, where higher sum score on the scale indicates higher levels of global self-esteem (range, 10–40).

### Depression

Depression was assessed using the moods and feelings questionnaire (MFQ; Costello & Angold, 1988). The MFQ is a 33-item scale used as a screening tool for depression in children and young people. It has demonstrated good validity and reliability in both clinical and non-clinical samples (Thabrew, Stasiak, Bavin, Frampton, & Merry, 2018).

### Attention deficit hyperactivity disorder

ADHD symptoms were assessed with the adult ADHD self-report scale (ASRS) part A screener (Kessler et al., 2007). This is a six-item questionnaire that includes questions on inattention (four items) and hyperactivity symptoms (two items) in the previous six months. Participants responded using a five-point response scale with answer options ranging from ‘never’ – scored 0 to ‘very often’ – scored 4. Responding ‘sometimes’, ‘often’ or ‘very often’ to at least four questions reflects the presence of symptoms consistent with ADHD (Kessler et al., 2007). Based on previous work (Blanco et al., 2009), factor analysis was conducted using varimax rotation to extract scores for inattention and for hyperactivity-impulsivity on the instrument.

### Trait impulsivity

The BIS, brief version (BIS-Brief) was used to measure unidimensional trait impulsivity. The BIS-Brief is an eight-item self-report questionnaire with good reliability and validity in both healthy young people and patients (Mathias et al., 2018).

### Trait impulsivity (measured previously)

The BIS, version 11 (BIS-11), was used to measure trait impulsivity: attentional impulsivity, motor impulsivity, and non-planning impulsivity. The BIS-11 is a 30-item self-report questionnaire shown to have good reliability and validity in both clinical and non-clinical subjects (Stanford et al., 2009). This questionnaire was collected on average 2.8 years prior to the above measures, in a preceding data round.

### Disordered eating

The SCOFF questionnaire was used to assess disordered eating behaviour (Hill, Reid, Morgan, & Lacey, 2010). The SCOFF is not a diagnostic instrument but has been found to have high specificity and sensitivity (Luck et al., 2002). It comprises five questions (yes/no response format) asking whether in the past year the participant (1) had lost more than one stone (6.35 kg) in three months (weight loss), (2) had made him/herself be sick because he/she felt uncomfortably full (self-sick for feeling full), (3) worried that he/she had lost control over how much he/she eats (uncontrolled eating), (4) believed him/herself to be fat when others said that he/she was too thin (self-perceived fatness), and (5) thought that food dominated his/her life (food dominance). Responding yes to at least two items was considered a positive screen for possible ED (Morgan, Reid, & Lacey, 1999). The SCOFF is not a diagnostic instrument but has been found to have high specificity and sensitivity for detecting AN and BN. However, it may be less effective in identifying binge eating disorder (Solmi, Hatch, Hotopf, Treasure, & Micali, 2015). It has high negative predictive validity but more moderate positive predictive validity, which is common for low prevalence conditions (Luck et al., 2002; Solmi et al., 2015). A recent meta-analysis of 25 studies reported the validity of the SCOFF to be high across samples with a pooled sensitivity of 0.86 [95% confidence interval (CI) 0.78–0.91] and specificity of 0.83 (95% CI 0.77–0.88). The test re-test reliability has been shown to be high (Garcia et al., 2011; Garcia-Campayo et al., 2005).

### Data processing and analysis

High and low symptom groups were compared on demographic variables and potential covariates using *t*-tests. Groups of participants were assigned to high (*n* = 167) or low ADHD (*n* = 475) symptoms based on the recommended cut-off for symptom scoring consistent with ADHD in adults provided in the ASRS.

Three hierarchical regressions (two for the cross-sectional and one for the longitudinal relationship) were conducted. Both the cross-sectional and longitudinal model consisted of three hierarchical model steps. The outcome variable was eating disorder risk score. Participants were assigned to either a ‘high’ or ‘low’ risk for eating disorders group, based on responses to the SCOFF questionnaire. Guidelines for using the SCOFF (Hill et al., 2010) suggest that a score of ≥2 reflects potential eating disorder risk. In the first step of the first cross-sectional model BIS-Brief scores were entered, and in the second cross-sectional regression model ASRS inattentive and hyperactive/impulsive scores were entered simultaneously. In the first step of the longitudinal regression model, the three BIS subscales (attentional impulsivity, motor impulsivity and non-planning impulsivity) were entered simultaneously. In the second step of all models age, sex and ethnicity were entered. In the third step for all models, MFQ scores, RSE scores, FAST scores, FTND scores, and illicit drug use scores were entered. Based on results from logistic regression analyses, mediation models were tested to assess the potential mediating influence of significant predictor variables.

Further exploratory analyses were conducted, consisting of hierarchical regression models, with each SCOFF subscale as the outcome variable in a separate regression model, and separate model steps described as above. These models were run only for predictor ASRS scores/BIS subscales that significantly predicted SCOFF risk status in the first regression. Bonferroni correction was applied to these models to account for multiple comparisons (*α* = 0.01).

## Results

### Participant characteristics

The sample consisted of 642 participants (mean age 23 years (± 3.1), 65% female). See Table 1 for participant characteristics. The prevalence of the possible eating disorder in the sample was 21.5%, 76% of whom were female. The prevalence of possible ADHD in the sample was 26%, 56% of whom were female. Participants scoring higher for disordered eating had higher: FTND, ASRS inattention and hyperactivity/impulsivity and higher BIS attentional impulsivity and non-planning impulsivity (see Table 1 for descriptive statistics).

**Table 1.** Group (ED risk/no ED risk) differences in questionnaire measures of alcohol use, smoking, illicit drug use and disordered eating

	All (*n* = 642)	SCOFF no risk (*n* = 504)	SCOFF ED risk (*n* = 138)			
Sex	416:223:3 (female:male:other)	311:191:2 (female:male:other)	105:32:1 (female:male:other)			
Ethnicity	74.6% White 7.5% Asian/British Asian 6.9% multiracial 4.4% Black/African/Caribbean 0.9% other 5.7% not recorded					
	Mean (s.d.)			*F*	*p*	np^2^
Age (years)	23.4 (3.1)	23.4 (3.1)	23.2 (3.0)	0.7	0.41	0.001
Alcohol use (FAST score)	6.3 (2.3)	6.3 (2.2)	6.6 (2.5)	1.8	0.18	0.003
Smoking (FTND score)	0.95 (2.8)	0.8 (2.6)	1.4 (3.3)	5.0	0.03	0.008
Illicit drug use	1.8 (0.4)	1.9 (0.4)	1.8 (0.4)	3.0	0.09	0.005
ASRS inattention score	5.2 (2.7)	4.7 (2.5)	6.7 (2.5)	68.7	<0.001	0.97
ASRS hyperactivity/impulsivity score	3.15 (1.8)	3.0 (1.7)	3.9 (1.9)	30.1	<0.001	0.05
BIS attentional impulsivity	15.2 (4.1)	14.8 (3.9)	16.9 (4.2)	24.8	<0.001	0.044
BIS motor impulsivity	20.6 (4.2)	20.5 (4.3)	21.1 (4.0)	1.5	0.22	0.003
BIS non-planning impulsivity	23.9 (5.2)	23.6 (5.2)	25.0 (4.9)	7.2	0.008	0.013

ED risk = responding ‘yes’ to at least two SCOFF questions.

### Cross-sectional analysis

#### Cross-sectional logistic regression: BIS-Brief

The first model including BIS-Brief score only was statistically significant, χ^2^ (1) = 22.9, *p* < 0.001 and explained 6% (Nagelkerke *R*-square) of the variance associated with being classified as ‘at risk’ and 80% of cases were classified correctly. The second model step (additionally including age, sex, and ethnicity) significantly improved model fit χ^2^ (3) = 10.39, *p* = 0.02. BIS-Brief score remained a significant predictor in this model (*β* = 1.4, *p* < 0.001, Exp(*B*) = 1.15) and sex also significantly contributed to the model (*β* = 0.7, *p* = 0.002, Exp(*B*) = 2.1). This model explained 9% (Nagelkerke *R*-square) of the variance associated with being classified as ‘at risk’ and 80% of the cases were correctly classified. The third model step (additionally including MFQ, RSE, FAST, FTND, and illicit drug use) significantly improved model fit χ^2^ (5) = 32.1, *p* < 0.001. BIS-Brief score (*β* = 0.1, *p* = 0.001, Exp(*B*) = 1.1) and sex (*β* = 0.7, *p* = 0.005, Exp(*B*) = 2.0) remained significant predictors in the model, and MFQ significantly contributed to the model (*β* = 0.4, *p* < 0.001, Exp(*B*) = 1.0). The final model was statistically significant χ^2^ (9) = 65.3, *p* < 0.001, explained 17% (Nagelkerke *R*-square) of the variance associated with being classified as ‘at risk’ and 81% of cases were classified correctly.

#### Cross-sectional logistic regression: ASRS

The first model including ASRS factors (hyperactive/impulsive and inattentive symptoms) was statistically significant, χ^2^ (2) = 47.7, *p* < 0.001 and explained 13% (Nagelkerke *R*-square) of the variance associated with being classified as ‘at risk’ and 79% of cases were classified correctly. Both of the two ASRS factors individually significantly contributed to the model: inattentive symptoms (*β* = 0.233, *p* < 0.001, Exp(*B*) = 1.3), hyperactive/impulsive symptoms: *β* = 0.13, *p* = 0.05, Exp(*B*) = 1.1. The second model step (additionally including age, sex, and ethnicity) significantly improved model fit χ^2^ (3) = 10.42, *p* = 0.02. This model explained 15% (Nagelkerke *R*-square) of the variance associated with being classified as ‘at risk’ and 80% of the cases were correctly classified. The third model step (additionally including MFQ, RSE, FAST, FTND, and illicit drug use) significantly improved model fit χ^2^ (5) = 20.0, *p* = 0.001. The contribution of inattentive symptoms and sex remained significant in the model, and MFQ scores significantly contributed to the model. hyperactive/impulsive symptoms no longer significantly contributed to the model (see Table 2). Inattentive symptoms of ADHD associated with all individual ED symptoms with the risks apart from weight loss: self-sick for feeling full (*β* = 0.327, *p* < 0.001, Exp(*B*) = 1.387), uncontrolled eating (*β* = 1.46, *p* = 0.003, Exp(*B*) = 1.15), self-perceived fatness (*β* = 0.177, *p* = 0.004, Exp(*B*) = 1.19), food dominance (*β* = 0.163, *p* = 0.002, Exp(*B*) = 1.17), and weight loss (*β* = 0.012, *p* = 0.96, Exp(*B*) = 0.99). In contrast, hyperactive/impulsive symptoms were associated with uncontrolled eating only (*β* = 1.58, *p* = 0.03, Exp(*B*) = 1.17).

**Table 2.** Summary of binary logistic regression statistics for the cross-sectional analysis (model 3)

	*B*	Sig.	Exp(*B*)	95% CI lower for Exp(*B*)	95% CI upper for Exp(*B*)
ASRS att.	0.194	<0.001	1.2	1.1	1.3
ASRS hyp/imp.	0.088	0.23	1.09	0.95	1.3
Age	−0.5	0.22	0.95	0.88	1.03
Sex	0.71	0.006	2.03	1.2	3.4
Ethnicity	0.031	0.81	1.03	0.81	1.3
Mood and feelings	0.033	<0.001	1.03	1.02	1.05
Rosenberg	0.077	0.22	1.08	0.96	1.2
FAST	0.45	0.4	1.05	0.94	1.2
FSTROM	−0.2	0.63	0.98	0.91	1.06
Illicit drug	−0.24	0.40	0.78	0.45	1.4

Control variables/covariates are shaded grey and variable of interest are light blue

#### Mediation

**Fig. 1.**
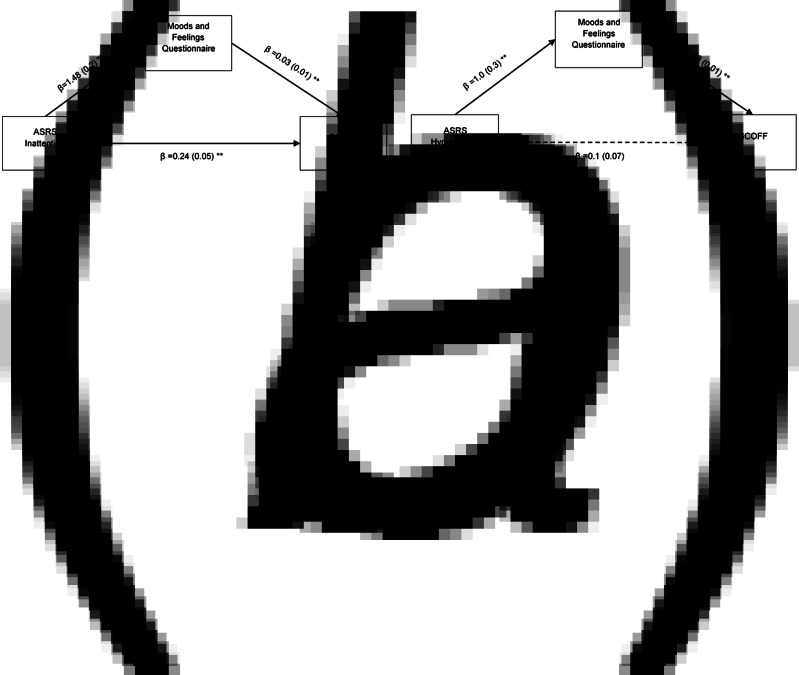
Cross-sectional mediation models. (a) The model shows the relationship between ASRS inattentive symptoms and SCOFF risk. (b) The model shows the relationship between ASRS hyperactive/impulsive symptoms and SCOFF risk. Solid lines reflect significant pathways. Estimates (*β*) are unstandardised regression coefficients, numbers in parentheses show bootstrapped standard error. All analyses controlled for sex, alcohol use, smoking, illicit drug use, and age. **p < 0.001.

Inattentive symptoms (controlling for hyperactive symptoms) predicted SCOFF risk category both directly (Effect = 0.22, s.e. = 0.05, *Z* = 4.5, *p* < 0.001, CI 0.12–0.313) and indirectly through MFQ scores (Effect = 0.051, Bootstrapped CI 0.027–0.82) (see Fig. 1*a*). Hyperactive/impulsive symptoms (controlling for inattentive symptoms) did not predict SCOFF risk category directly (Effect = 0.01, s.e. = 0.07, *Z* = 1.4, *p* = 0.17, CI −0.042 to 0.24) but did predict SCOFF risk category indirectly through negative mood (Effect = 0.04, Bootstrapped CI 0.013–0.07) (see Fig. 1*b*). Because women were more likely than men to be at risk for disordered eating we examined sex as a moderating variable. Sex did not moderate the relationship between ADHD symptoms and mood and feelings scores nor the relationship between MFQ and SCOFF risk category (inattention: index = 0.04, bootstrapped CI 0.00–0.096, hyperactivity/impulsivity: index = −0.0004, bootstrapped CI −0.035 to 0.034).

### Longitudinal analysis

#### Longitudinal logistic regression: BIS subscales

The first model including BIS subscales (attentional impulsivity, motor impulsivity, and non-planning impulsivity) was statistically significant, χ^2^ (3) = 21.97, *p* < 0.001 and explained 7% (Nagelkerke *R*-square) of the variance associated with being classified as ‘at risk’ and 82% of cases were classified correctly. Attentional impulsivity was the only BIS subscale to significantly contribute to the model (*β* = 1.4, *p* < 0.001, Exp(*B*) = 1.15). The second model step (additionally including age, sex, and ethnicity) did not significantly improve model fit χ^2^ (3) = 5.0 and *p* = 0.172. This model explained 9% (Nagelkerke *R*-square) of the variance associated with being classified as ‘at risk’ and 82% of the cases were correctly classified. The third model step (additionally including MFQ, RSE, FAST, FTND, and illicit drug use) significantly improved model fit χ^2^ (5) = 20.0 and *p* = 0.001. The contribution of attentional impulsivity and sex remained significant in the model, and MFQ scores significantly contributed to the model (see Table 3). The motor impulsivity and non-planning impulsivity subscales were not associated with disordered eating risk (*p* = 0.58 and *p* = 0.99, respectively). Attentional impulsivity associated specifically with uncontrolled eating (*β* = 0.124, *p* = 0.001, Exp(*B*) = 1.132) and self-perceived fatness (*β* = 0.11, *p* = 0.008, Exp(*B*) = 1.123) but not self-sick for feeling full (*β* = 0.120, *p* = 0.032, Exp(*B*) = 1.127), weight loss (*β* = −0.013, *p* = 0.825, Exp(*B*) = 0.938), or food dominance (*β* = 0.048, *p* = 0.2 Exp(*B*) = 1.05).

**Table 3.** Summary of binary logistic regression statistics for the longitudinal analysis (model 3)

	*B*	Sig.	Exp(B)	95% CI lower for Exp(*B*)	95% CI upper for Exp(*B*)
BIS attention	0.078	0.045	1.1	1.0	1.2
BIS motor	−0.02	0.58	0.98	0.92	1.05
BIS non-planning	0.00	0.99	1.0	0.94	1.06
Age	−0.004	0.93	1.0	0.92	1.08
Sex	0.64	0.024	1.9	1.09	3.3
Ethnicity	−0.01	0.93	0.99	0.75	1.3
Mood and feelings	0.038	<0.001	1.04	1.02	1.06
Rosenberg	0.087	0.20	1.1	0.95	1.2
FAST	0.089	0.14	1.1	0.97	1.2
FSTROM	−0.002	0.96	1.0	0.91	1.09
Illicit drug	−0.25	0.45	0.78	0.42	1.5

Control variables/covariates are shaded grey and variable of interest are light blue

#### Mediation

**Fig. 2.**
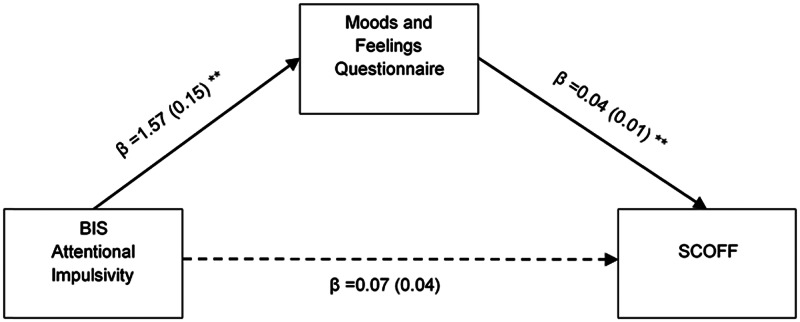
Longitudinal mediation model. Solid lines reflect significant pathways. Estimates (*β*) are unstandardised regression coefficients, numbers in parentheses show bootstrapped standard error. All analyses controlled for sex, alcohol use, smoking, illicit drug use, and age. **p < 0.001.

Because there was no relationship between BIS motor impulsivity and non-planning impulsivity and disordered eating risk the mediation analysis focused only on BIS attentional impulsivity. Attentional impulsivity did not predict SCOFF risk category directly (Effect = 0.07, s.e. = 0.04, *Z* = 1.8, *p* = 0.06, CI −0.003 to 0.15) but did predict SCOFF risk category indirectly through scores on the MFQ (Effect = 0.07, Bootstrapped CI to 0.038–0.1) (See Fig. 2). Because women were more likely than men to be at risk for disordered eating we examined sex as a moderating variable. Sex did not moderate the relationship between ADHD symptoms and MFQ scores nor the relationship between MFQ scores and SCOFF risk category (Index = −0.01, Bootstrapped CI −0.04 to 0.012).

## Discussion

We observed a positive cross-sectional relationship between inattentive symptoms of ADHD and overall risk of disordered eating in a community sample of young adults, even when controlling for a range of covariates. We also observed a prospective relationship between trait impulsivity and risk of disordered eating in the same sample. The association between trait impulsivity and eating disorder risk was fully mediated by depression scores, whereas the association between inattentive symptoms and eating disorder risk was only partially mediated by depression. None of the relationships between ADHD symptoms and trait impulsivity were moderated by sex, suggesting that they hold for both young men and women.

In the cross-sectional analysis, inattentive symptoms of ADHD were associated directly both with overall eating disorder risk and all individual risk categories of the SCOFF (self-sick, self-perceived fatness, food dominance, and uncontrolled eating) apart from weight loss. Hyperactive/impulsive symptoms were not associated with overall risk in the final model but did predict uncontrolled eating. Although depression scores fully mediated the association between hyperactive/impulsive symptoms and uncontrolled eating risk, the relationship between inattentive symptoms and overall eating disorder risk was only partially mediated by depression scores. These findings are in agreement with the results of Kaisari et al. (2018) who found that inattentive symptoms of ADHD (as assessed by the Conners adult rating scale – CAARS; Conners et al., 1999) were directly and indirectly associated with both binge-disinhibited and restrictive eating, whereas hyperactive/impulsive symptoms were more consistently associated with binge-disinhibited eating and this relationship was mediated by negative mood. Taken together, these data suggest that inattentive symptoms are directly associated with a range of disordered eating symptoms independent of negative mood whereas hyperactive-impulsive symptoms appear to be mainly indirectly associated with binge/uncontrolled eating via depressive symptoms.

It is possible that binge/uncontrolled eating provides a mechanism for coping with the negative affect that is associated with experiencing ADHD symptoms. Depression and mood disorders are often co-morbid with ADHD (Friedrichs, Igl, Larsson, & Larsson, 2012) and depressive symptoms have been reported to predict binge eating (Spoor et al., 2006), which is consistent with the suggestion from the present data that the association between ADHD symptoms (particularly hyperactive/impulsive symptoms) and uncontrolled eating is likely to be explained by depressive symptoms. However, the finding that depression scores did not fully mediate the association between inattentive symptoms of ADHD and eating disorder risk suggests that there are additional underlying mechanisms that could account for this relationship. Indeed, our regression models only explained around 20% of the variance in disordered eating risk. Jacob, Haro, and Koyanagi (2018) observed a positive cross-sectional relationship between ADHD scores and disordered eating risk in a nationally representative sample and found that between 28% and 42% of the association between ADHD symptoms and possible eating disorder was explained by stressful life events, anxiety, and borderline personality traits, which we did not measure in this study but could have similarly accounted for at least some of the additional variance. In addition, there could be a distinct contribution of factors related specifically to inattentive symptoms of ADHD that act independently of co-morbidities.

For example, inattentive symptoms (which include forgetfulness and difficulty in organising tasks) are linked to the impaired perception of and/or utilisation of interoceptive signals to guide appetitive behaviour, which in turn is related to increased risk of disordered eating (Martin, Dourish, Rotshtein, Spetter, & Higgs, 2019). In support of this suggestion, Kaisari et al. (2018) reported that inattentive symptoms of ADHD were associated with decreased awareness of internal signals of hunger and satiety, and in turn, these deficits were positively associated with disordered eating. Another alternative mechanism is that inattentive symptoms may be associated with the impaired encoding of episodic food memories, which has been shown to influence subsequent responses to food cues (see Higgs, 2016 and Higgs et al., 2017 for a review). For example, distraction while eating (e.g. watching TV) has been shown to impair the memory encoding of a meal and result in an increase in subsequent snack intake. It is, therefore, possible that individuals with pronounced inattentive symptoms of ADHD may be easily distracted when eating, resulting in impaired memory for recent eating, leading to subsequent overeating, especially in the presence of highly palatable foods. Interestingly, problems with episodic memory more generally have been associated with overeating and obesity (Cheke, Simons, & Clayton, 2016; Higgs & Spetter, 2018; Higgs, Williamson, Rotshtein, & Humphreys, 2008). While the role of depression and anxiety in the emergence of disordered eating has been appreciated for some time, how cognitive problems related to attention might contribute has received far less attention. Future research should further investigate the role of interoception and memory/attention processes as mediators of the association between inattentive symptoms of ADHD and eating disorder risk.

Longitudinally we observed that the trait of attentional impulsivity (as assessed by the BIS) predicted risk of eating disorder indirectly through negative mood. This finding is consistent with the results of previous cross-sectional studies that have reported an association between impulsivity and eating disorders (e.g. Lee-Winn, Townsend, Reinblatt, & Mendelson, 2016) and with our cross-sectional finding that hyperactive/impulsive symptoms were associated with risk of eating disorder via depression. Few studies to date have examined the longitudinal association between trait impulsivity and eating disorder risk in general populations (especially young adult populations) and most studies have not distinguished between different facets of impulsivity nor examined the mediating mechanisms (Bénard et al., 2019; Bodell, Joiner, & Ialongo, 2012; Evans et al., 2019; Mikami et al., 2010; Pearson, Combs, Zapolski, & Smith, 2012). Here, we find that the attentional impulsivity, but neither non-planning nor motor impulsivity predicted eating disorder risk longitudinally via depression scores. From the present data, it is not clear why the association between trait impulsivity and eating disorder risk should be specific to attentional impulsivity but one possibility is that an underlying factor relating to both trait attentional impulsivity and inattentive symptoms of ADHD explains the relationship. Questions from the BIS assessing trait impulsivity ask about difficulty concentrating and having extraneous thoughts as well as ability to ‘pay attention’, which could affect eating behaviours via the effects of distractibility on interoception and memory processes outlined above and reported previously (Kaisari et al., 2018). Hence, our findings are suggestive of an important role for attentional mechanisms generally in the development of disordered eating.

Attentional impulsivity may also enhance appetitive responses to food cues which could promote binge/overeating tendencies. In support of this suggestion, a review of the relationship between BIS subscales and measures of eating behaviour suggested that attentional impulsivity was most consistently related to the tendency towards overeating, whereas the relationship with motor impulsivity was less consistent and was very weak for non-planning impulsivity (Meule, 2013). Moreover, attentional impulsivity in binge eating disorder patients has been shown to be related to poorer response inhibition to food cues and hypoactivity in the prefrontal cognitive control network that regulates responsiveness to food cues (Hege et al., 2015).

We found that impulsivity, as assessed by the ASRS, was not associated with overall eating disorder risk (although it was associated with uncontrolled eating) in the cross-sectional analysis, whereas impulsivity, as assessed by the BIS, predicted overall risk in the longitudinal analysis. The reason for this probably relates to the fact that the ASRS measure captures both impulsiveness and hyperactivity, whereas the BIS focuses in more detail on impulsivity rather than hyperactivity. Further work is required to assess the specific contribution (if any) of hyperactive symptoms to eating disorder risk and to tease out which components of impulsive behaviour are important.

Particular strengths of the present study are that the sample size is relatively large and represents a normative cohort including young women and men from whom the conclusions are broadly generalisable. In line with previous studies, we did not find that sex was a moderating influence on any of the relationships between ADHD symptoms and eating disorder risk (Brewerton & Duncan, 2016; Kaisari et al., 2018). We also controlled for several covariates in our analyses to account for the potential confounding effects of factors such as age and co-morbid drug use. Along with these strengths, the findings should also be interpreted within the context of some limitations. Because the ASRS was only included in the follow-up assessment period we were unable to conduct a longitudinal analysis on these data. In addition, the longitudinal analysis involved only two assessment points which means that causal inferences from the mediational findings cannot be made. Further studies using at least three assessment points are required to tease out the temporal associations between impulsivity and depression in predicting disordered eating risk. We also note that it has been suggested that hyperactive-impulsive symptoms change at different rates over time in ADHD (for discussion see Martel, Levinson, Langer, & Nigg, 2016), and this may have differentially impacted the statistical ability to detect longitudinal relationships with other variables. In addition, the prevalence of possible ADHD in our sample was much higher than the reported prevalence of ADHD in the general population when estimated based on clinical diagnosis. In this context, it is important to consider that the ASRS was developed as a screener for ADHD and is not a diagnostic tool. Thus, while it has good negative predictive value, the positive predictive value is more moderate and therefore could over-identify ADHD. However, over-identifying ADHD would not explain the association between ADHD symptoms and possible eating disorder and indeed might be expected to mask any group differences that exist. Finally, the SCOFF screener only identifies participants with a possible eating disorder rather than a clinical diagnosis. However, disordered eating patterns which do not meet clinical criteria are, nevertheless, often associated with psychopathology (Tanofsky-Kraff, Engel, Yanovski, Pine, & Nelson, 2013).

In conclusion, in a normative cohort of young men and women, we found that inattentive symptoms of ADHD were directly associated with increased risk of an eating disorder and measures of impulsivity were associated indirectly via depression with the risk of an eating disorder both cross-sectionally and longitudinally. These data highlight the need for further investigation of the specific role that inattentive symptoms play in the development of eating disorders. Our findings also suggest that future research should be directed towards unravelling the relationships between depression and impulsivity in predicting the emergence of eating disorders. A better understanding of the role that ADHD-related symptoms have in the risk for developing eating disorders will inform future prevention, detection and treatment strategies. For example, the presence of inattentive and impulsive symptoms as well as depression could be assessed as indicators of enhanced risk of the development of eating disorders. In addition, clinical management for individuals with ADHD and eating disorders might benefit particularly from the treatment of psychiatric comorbidities such as depression. Finally, future research should assess whether treatments and behavioural therapies that directly target attentional deficits may prove effective in the management of both ADHD and eating disorders.
